# Misoplegia and dementia: a case study

**DOI:** 10.1590/1980-57642021dn15-020019

**Published:** 2021

**Authors:** Diogo Haddad Santos, Yngrid Dieguez Ferreira, Gilvan Guersoni Hora, Luiza Ramos de Freiras, Paulo Henrique Maia de Freitas, Bruna Garbugio Dutra, Rubens José Gagliardi

**Affiliations:** 1 Irmandade da Santa Casa de Misericórdia de São Paulo - São Paulo, SP, Brazil.; 2 School of Medical Sciences, Santa Casa de São Paulo- São Paulo, SP, Brazil.

**Keywords:** dementia, neurodegenerative disease, mental disorders, self-injurious behavior, demência, doenças neurodegenerativas, transtorno do comportamento, comportamento autodestrutivo

## Abstract

Brain-damaged patients can develop abnormal attitudes towards their deficits. Misoplegia is one such example, involving exaggerated aversion to an impaired limb, sometimes associated with hatred of paresis and verbal or physical abuse directed at the paretic limb. Few studies or reports on this disorder are available in the literature, prompting the present case report of a patient with misoplegia and vascular dementia.

## RESUMO.

Pacientes com lesões cerebrais podem apresentar atitudes anormais em relação a seus déficits. Um exemplo é a misoplegia, uma aversão excessiva em relação ao membro com déficit, podendo estar associado a ódio à paralisia e maus tratos verbais ou físicos contra os membros paralisados. Ainda há poucos estudos e relatos sobre esse distúrbio na literatura, evidenciando a importância de um relato de caso de um paciente portador de misoplegia e demência vascular.

## INTRODUCTION

There is a fine line between diseases of the neurological and psychiatric spectrum. Numerous areas of the brain, when structurally damaged, can cause abnormal behavior which supports this assumption. The parietal lobe, for example, sometimes shows symptoms related to somatic awareness, often confounded with depressive disorders and even dementia. In 1914, Joseph Babinski was first to describe in patients the lack of awareness about their neurological deficit or simple failure to recognize their disease, he and defined it as anosognosia.^1^

Over time, neurological clinical experience has confirmed that hemiplegic patients, particularly those with right-hemisphere lesions, can exhibit abnormal attitudes regarding recognition of their deficits. These cases can include an insistence that the paretic limb belongs to someone else (somatoparaphrenia) and a total lack of awareness of the affected body side (neglect). In rare cases, exaggerated aversion to the impaired limb involving hatred of paresis or verbal and physical abuse directed at paretic limbs can occur, a condition defined as misoplegia.^2^ Few reports and studies on misoplegia are available in the literature.^3^ The present study provides a discussion of an illustrative case together with a literature review on the theme.

## CASE REPORT

We report the case of a 57-year-old woman who was left-handed, illiterate, had systemic arterial hypertension, diabetes mellitus and hypothyroidism, was a former smoker, and had no family history of known diseases or neuropsychiatric conditions. According to family informants, she had always been a calm, good-natured person.

The patient was admitted to the emergency department of a private hospital with sudden right hemiparesis and aphasia. A cranial computed tomography scan disclosed stroke in the left middle cerebral artery territory. Intravenous thrombolysis treatment was not performed owing to the time window. She was discharged after a 3-day hospital stay and started on secondary prophylaxis for further stroke.

A couple of months after the neurovascular event, the patient exhibited an onset of behavioral changes and cognitive decline and was referred to the center for cognitive disorders. According to reports by family members, the patient had become dependent on daily living activities, mainly due to the motor sequelae. However, she manifested irritability and hetero-aggressive behavior, with isolated episodes of swearing and self-injurious behavior involving the paretic limb. The general examination revealed scars produced by scratches, cuts and bites in upper and lower limbs on the right side of the body. On neurological examination, the patient exhibited marked cognitive impairment, with spontaneous attention deficit, temporospatial disorientation and limited thought content. She scored 10 out of 30 on the Mini-Mental State Examination and showed major dependence on the Functional Scales of Katz and Lawton. The examination also showed marked non-fluent aphasia with unsatisfactory comprehension and adequate repetition, besides disproportionate hemiparesis predominantly affecting the groin/thigh region and gait, along with swearing and insults to the paretic side and its deficits. Impaired comprehension precluded the application of tests for anosognosia, asomatognosia, neglect and left-right orientation. Similarly, other cognitive tests could not be applied owing to the patient´s low level of understanding. Consequently, a basic etiological assessment of vascular conditions realized with a non-invasive neuroimaging of vessels was done. The patient was maintained on antiplatelet drugs and statins, and besides, she was guided on the correct use of medication for adequate control of blood pressure and glycemia. An atypical neuroleptic was prescribed to control behavioral symptoms.

**Figure 1.**
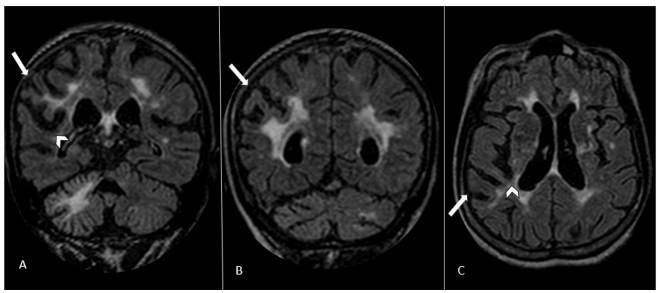
Flair sequences of brain MRI. Coronal (A and B) and axial (C) planes demonstrate asymmetric cerebral atrophy, with a right parietal lobe predominance. The right inferior parietal lobule (full arrow) and homolateral posterior perisylvian region (arrowhead) had a reduced cortical and subcortical volume, most notably in the right angular and supramarginal gyri. Extensive hyperintensity is depicted in the white matter of cerebral hemispheres and right cerebellum, with bilateral ischemic infarction lacunes in nucleocapsular region and cerebral hemispheres, possibly secondary to small vessel disease.

Brain magnetic resonance imaging (MRI) and cervical and cranial MR angiography (MRA) were performed nine months post-stroke. MRI showed confluent white matter cerebral lesions related to small vessel disease and asymmetric brain atrophy with a right parietal predominance (Figure 1). MRA demonstrated tortuosity of the intracranial arteries, albeit without stenosis. Transthoracic echocardiogram and electrocardiogram were unremarkable. The patient currently shows partial improvement of episodes and a stable cognitive picture, continuing to receive follow-up and specific care.

## DISCUSSION

An estimated 64% of older adults who suffer a stroke have developed some resultant cognitive impairment. On the other hand, a quarter of individuals with some degree of cognitive impairment have a stroke at some point.^4^ Therefore, patients with stroke-induced behavioral symptoms often present at referral centers for investigation of cognitive disorders or treatment for degenerative conditions. Concerted care should be exercised to differentiate between deficits secondary to the stroke itself and those associated with progressive neurodegenerative impairment triggered by the stroke event.

In a German study, 15% of all patients with strokes in the right-hemisphere associated with resulting hemiplegia had abnormal attitudes in terms of perceptions of the paretic limb, 25% of whom had some degree of misoplegia.^5^ The right posterior insular cortex and right parietal lobe appear to play a fundamental role in limb self-awareness.^6^ There are few typical cases reported in the literature, most of them with hemiplegia^7^ and very few involving children.^8^ Furthermore, no case report was found in which patients had dominance in the right hemisphere or in which lesions on the left brain side caused the right misoplegia. It suggests that the symptom is independent of functional lateralization.

Misoplegia is typically associated with brain lesions to the right side. Patients with a lesion on the same side can also sometimes confabulate, show neglect, have a phobia of images and objects presented to their left (levophobia),^9^ or even believe these are in another place (reduplicative paramnesia).^10^ Interestingly, the patient in our case was left-handed, displayed symptoms consistent with misoplegia in the right arm, and showed right hemisphere lesions, notwithstanding her neurological dominance.

For some time, scholars have posited previous personality traits as an important factor associated with possible inadequate behavioral changes in response to changes in body functioning. This definition may involve a previous concern with body effectiveness and physical aptitude, originally reported by Critchley,^2^ or may be attributed to a distorted body image associated with the acquired deficit. The current case exhibited no previously known psychological characteristics supporting these theories.

As demonstrated, many patients with lesions associated with changes in body recognition are treated as dementia cases, where some are given inappropriate vascular treatment to prevent further stroke episodes. Indeed, the explanation for these dysfunctions involving inadequate behavior for self-perceived body perception raises more questions than answers.

## References

[1] Babinski J (1914). Contribution to the study of mental disturbances in organic cerebral hemiplegia (anosognosia). Rev Neurol.

[2] Critchley M (1974). Misoplegia, or hatred of hemiplegia. Mt Sinai J Med.

[3] Fredericks J, Vinken PJ, Bruyn GW, Klawans HL, Fredericks JAM (1985). Disorders of the body schema. Handbook of clinical neurology.

[4] Jin YP, Di Legge S, Ostbye T, Feightner JW, Hachinski V (2006). The reciprocal risks of stroke and cognitive impairment in an elderly population. Alzheimers Dement.

[5] Baier B, Karnath HO (2008). Tight link between our sense of limb ownership and self-awareness of actions. Stroke.

[6] Cereda C, Ghika J, Maeder P, Bogousslavsky J (2002). Strokes restricted to the insular cortex. Neurology.

[7] Loetscher T, Regard M, Brugger P (2006). Misoplegia: a review of the literature and a case without hemiplegia. J Neurol Neurosurg Psychiatry.

[8] Moss AD, Turnbull OH (1996). Hatred of the hemiparetic limbs (misoplegia) in a 10 year old child. J Neurol Neurosurg Psychiatry.

[9] Bisiach E, Block N, Flanagan O, Güzeldere G (1997). Understanding Consciousness: clues from unilateral neglect and related disorders. The nature of consciousness: philosophical debates.

[10] Lee K, Shinbo M, Kanai H, Nagumo Y (2011). Reduplicative paramnesia after a right frontal lesion. Cogn Behav Neurol.

